# Effect of bowel preparation volume in inpatient colonoscopy. Results of a prospective, randomized, comparative pilot study

**DOI:** 10.1186/s12876-020-01373-1

**Published:** 2020-07-13

**Authors:** Patricia V. Hernandez, Jennifer L. Horsley-Silva, Diana L. Snyder, Noemi Baffy, Mary Atia, Laura Koepke, Matthew R. Buras, Elisabeth S. Lim, Kevin Ruff, Sarah B. Umar, Sameer Islam, Francisco C. Ramirez

**Affiliations:** 1 Department of Gastroenterology and Hepatology, Mayo Clinic Arizona, 13400 E Shea Blvd, Scottsdale, AZ 85259 USA; 2 Department of Health Sciences Research, Division of Biomedical Statistics and Informatics, Mayo Clinic Arizona, Scottsdale, USA

**Keywords:** Colonoscopy, Bowel preparation, Inpatient colonoscopy: colonoscopy preparation

## Abstract

**Background:**

Inpatient status has been shown to be a predictor of poor bowel preparation for colonoscopy; however, the optimal bowel preparation regimen for hospitalized patients is unknown. Our aim was to compare the efficacy of bowel preparation volume size in hospitalized patients undergoing inpatient colonoscopy.

**Methods:**

This prospective, single blinded (endoscopist), randomized controlled trial was conducted as a pilot study at a tertiary referral medical center. Hospitalized patients undergoing inpatient colonoscopy were assigned randomly to receive a high, medium, or low-volume preparation. Data collection included colon preparation quality, based on the Boston Bowel Preparation Scale, and a questionnaire given to all subjects evaluating the ability to completely finish bowel preparation and adverse effects (unpleasant taste, nausea, and vomiting).

**Results:**

Twenty-five colonoscopies were performed in 25 subjects. Patients who received low-volume preparation averaged a higher mean total BBPS (7.4, SD 1.62), in comparison to patients who received high-volume (7.0, SD 1.41) and medium-volume prep (6.9, SD 1.55), *P* = 0.77. When evaluating taste a higher score meant worse taste. The low-volume group scored unpleasant taste as 0.6 (0.74), while the high-volume group gave unpleasant taste a score of 2.2 (0.97) and the medium-volume group gave a score of 2.1 (1.36), *P* < 0.01.

**Conclusion:**

In this pilot study we found that low-volume colon preparation may be preferred in the inpatient setting due its better rate of tolerability and comparable bowel cleanliness when compared to larger volume preparation, although we cannot overreach any definitive conclusion. Further more robust studies are required to confirm these findings.

**Trial registration:**

The Affect of Low-Volume Bowel Preparation for Hospitalized Patients Colonoscopies. Trial registration: NCT01978509 (terminated). Retrospectively registered on November 07, 2013.

## Background

Colonoscopy examinations are the standard test to evaluate the colon and are frequently performed in hospitalized patients for a number of indications [1]. The quality of colon cleansing directly affects the ability to visualize mucosa which herein affects diagnostic yield and ability to perform therapeutics [1, 2]. When bowel preparation is poor, it leads to significant limitations and prevents an endoscopist from performing a high quality examination. This may result in delay of the procedure or earlier interval colonoscopy, which increases cost and decreases patient safety [3–5].

Inpatient status has been shown to be a predictor of poor bowel preparation. This is thought to be due to patient characteristics such as older age, decreased mobility, and more comorbidities, in addition to the need for more emergent evaluation than outpatient populations [6, 7]. The ideal colon preparation method should empty the entire colon from fecal material in a rapid fashion, be as comfortable as possible for patient use, be associated with minimal adverse risks, and be cost efficient. Unfortunately, many of these features are not currently available in bowel preparation solutions [1, 4]. All colonoscopy preparation regimens may cause adverse effects such as electrolyte and fluid shifts, nausea, vomiting, and abdominal discomfort [8].

Studies in outpatient populations have demonstrated that timing and choice of cathartic medication affects the cleanliness of the bowel preparations [9–11]. However, no standardized (or optimized) bowel preparation regimen exists for inpatient populations undergoing colonoscopy. We hypothesized that a low-volume colon preparation regimen results in better quality of colon preparation in the inpatient setting when compared to traditional high or medium-volume regimens. The purpose of this study is to compare the efficacy of a bowel preparation size in hospitalized patients undergoing colonoscopies in the inpatient setting. Primary outcome measured was the quality of bowel preparation scored through the Boston Bowel Preparation Scale (BBPS). Secondary outcomes assessed were delay of procedure or cancellation due to poor bowel preparation, patient tolerance, and cecal intubation rate.

## Methods

### Trial design and setting

This prospective, single-blinded, randomized control trial (clinicaltrials.gov: NCT01978509) was approved by the Institutional Review Board (IRB) and adheres to Consolidated Standards for Reporting Trial (CONSORT) guidelines for reporting clinical trials. It was conducted as a pilot study at Mayo Clinic Arizona between September 2013 and March 2019.

### Study population

Eligible subjects included hospitalized patients aged 18 years or older who were able to provide consent and in whom colonoscopy was deemed medically necessary while hospitalized. Patients who were unable to give consent, were pregnant or breastfeeding, had renal impairment, ileus, ascites, toxic megacolon, evidence of gastrointestinal obstruction, or presence of an allergy to a study drug were excluded. Patients with toxic colitis, those who were unable to split the bowel preparation, those at risk for aspiration, those at risk for severe cardiac arrhythmias, and those who had a contraindication for bowel preparation were also excluded from the study. Risks and benefits were explained to all subjects and written informed consents were obtained.

### Bowel preparation

After informed consent was obtained, patients were randomly assigned to the high-volume solution polyethylene glycol (GoLYTELY®), the medium-volume solution polyethylene glycol + ascorbic acid (MoviPrep®) or low-volume solution sodium picosulfate (Prepopik®), see Table 1 for full list of ingredients. All doses were prescribed and administered as split dose, with half of the required preparation being administered the night before the procedure starting at six in the evening and the other half being administered the morning of the procedure starting at three in the morning. All patients were required to complete the liquid purgative 2 h prior to their procedure. These are further described in Table 1. All subjects received a clear liquid diet the day before the procedure.

**Table 1:** Bowel preparation regimens

**Prep Types**	**Volumes of Prep**	**Ingredients**	**Administration (full dose regimen)**
**Large volume preparation**	4000 mL	Polyethylene glycol, sodium sulfate, sodium bicarbonate, sodium chloride, potassium chloride	2 L-solution of water mixed to GoLYTELY® given in the evening before the colonoscopy. This regimen was repeated again the next morning
**Medium volume preparation**	2000 mL	Polyethylene glycol, sodium sulfate, sodium bicarbonate, sodium chloride, potassium chloride, sedum ascorbate and ascorbic acid	1 L-solution of water mixed to MoviPrep® given in the evening before the colonoscopy. This regimen was repeated again the next morning
**Low volume preparation**	300 mL	Sodium sulfate, potassium sulfate and magnesium sulfate	150 mL-solution of water mixed with Prepopik® given in the evening before colonoscopy. This regimen was repeated again the next morning

### Randomization

Physicians performing the endoscopy were blinded to what type of bowel preparation each patient received. Fellow physicians within the gastroenterology department on service at the hospital enrolled participants. The allocation ratio was 1:1:1 for the intervention. Randomization was carried out using a computer-generated random numbers model and performed by a nurse practitioner who then placed bowel preparation orders without informing the inpatient gastroenterology service or endoscopist(s) performing the colonoscopy. Additionally, the patients were told not to speculate or inform their nursing staff, physicians, or performing endoscopist(s) if they were aware which bowel preparation regimen they consumed. Both fellow physicians and faculty physicians completing the procedures were involved in the creation of the BBPS for formal reports and blinded to the preparation the patient received.

### Procedures

Colonoscopy procedures were performed by the inpatient gastroenterology hospital service, which included attending faculty and fellows (under direct supervision of an attending), using Olympus Exera II 180 series colonoscopes in 4 subjects and Olympus 190-series (CF-HQ190AL and PCF-H190L) colonoscopes in the remaining cases (Olympus Corp., Tokyo, Japan). The success of cecal intubation was established by visualization of anatomic landmarks (appendiceal orifice and ileocecal valve). Procedures were performed under conscious sedation (IV fentanyl, IV midazolam) in 21 subjects, while 4 patients underwent deep sedation (IV fentanyl, IV midazolam, and IV propofol) with assistance of anesthesia providers.

Therapeutic interventions, such as biopsies, polypectomies, clip placement, argon plasma coagulation, or other electrocoagulation modality were performed as indicated. During the colonoscopy, quality metrics (BPSS, and cecal intubation) were obtained. No procedures were required to be repeated due to inadequate preparation. Withdrawal time was not a quality metric tracked in this study due to inpatient procedures being performed for diagnostic purposes, not screening or surveillance.

### Primary outcome

To determine quality of bowel preparation among three different volume solutions, we compared the efficacy of low-volume bowel preparation to medium and high-volume preparation for bowel cleansing in hospitalized patients undergoing colonoscopy. We used the Boston Bowel Preparation Scale (BBPS) in the three colon segments (right, transverse and left colon) along with total score to determine quality.

### Secondary outcomes

Delay of procedure or cancellation due to poor bowel preparation was tracked, and cecal intubation rate was obtained. Tolerability was assessed via questionnaire. After colonoscopy, all subjects received a questionnaire about their experiences with the colonoscopy preparation (Table 2). The questionnaire given to patients included questions on percentage of bowel preparation completed, perceived unpleasant taste, symptoms of nausea and vomiting.

**Table 2:** Patient and procedure characteristics

**Variables**	**Data**
**Patient demographics**	• Sex • Age • BMI • Diabetes status • History of constipation • Indication for colonoscopy • Purgative type
**Colonoscopy features**	• Colon cleanliness quality, based on the Boston Bowel Preparation Scale • Cecal intubation
**Patient questionnaire**	• Ability to completely finish bowel preparation • Evaluation of adverse effects (unpleasant taste, nausea, and vomiting) using a five-point scale ranging from 0 (no symptoms) to 4 (severe symptoms)

### Statistical analysis

This was a pilot study, in which a modest sample size was achieved. The number of patients enrolled in this study was determined by willingness for participation. The trial ended due to a difficulty of recruiting participants, partly due to faculty and fellow bias that low volume colon preparation would lead to poor bowel preparation and need for repeat procedures.

Continuous variables are described with their mean and standard deviation while categorical variables are described by count and percentage. The Chi-Square test was used for demographics, indication for colonoscopy, diabetic status, history of constipation, purgative type, and if patient completed bowel preparation. The three groups were compared in terms of BMI, quality of bowel preparation (using Boston Bowel Preparation Scale), and side effects (unpleasant taste, nausea, vomiting, abdominal pain scored in a five-point scale) by the one-way analysis of variance (one-way ANOVA) test. All hypothesis tests were two-sided with *P* < 0.05 considered statistically significant. Analyses were performed using SAS 9.4 (SAS Institute, Inc.; Cary, NC).

## Results

Out of 32 initially randomized subjects, 7 patients did not complete the questionnaire after colonoscopy and therefore had incomplete data and were excluded. A total of 25 inpatient colonoscopies were performed in 25 subjects whom all had complete data. Nine patients were assigned to receive high volume preparation, eight received medium volume preparation, and eight received low volume preparation. Demographic data and underlying conditions such as diabetes and chronic constipation are reported in Table 3.

**Table 3:** Patient demographics and indication for colonoscopy

	**Large volume preparation (*n*** **= 9)**	**Medium volume preparation (*n*** **= 8)**	**Low volume preparation (*n*** **= 8)**	**Total** **(*n*** **= 25)**	***P*-value**
**Female gender, n (%)**	3 (33.3%)	3 (37.5%)	2 (25.0%)	8 (32.0%)	0.86
**Age (years)**					
Mean (±SD)	70.8 (±12.33)	62.1 (±17.46)	66.5(±19.79)	66.6 (±16.34)	0.57
Range	51–86	32–85	35–84	32–86	
**BMI (kg/m**^**2**^**)**					
Mean (±SD)	29.1 (±4.21)	25.3 (±3.67)	29.8 (±7.59)	28.2 (±5.57)	0.25
Range	22.9–35.7	18.2–29.8	23.2–45.6	18.2–45.6	
**Diabetes, n (%)**	2 (22.2)	2 (28.6)	2 (25.0)	6 (25.0)	0.96
**Chronic constipation, n (%)**	1 (11.1)	1 (14.3)	2 (25.0)	4 (16.7)	0.73
**Neurologic disease, n (%)**	0	Lewy body dementia: 1 (12.5)	Paraplegia: 1 (12.5)	2 (8)	0.36
**Use of medication that may cause constipation**	Opiate: 2 (22.2)	Opiate: 1 (12.5) Opiate + Carbidopa: 1 (12.5)	Opiate: 2 (25) Nortryptiline: 1 (12.5)	Opiate: 5 (20) Opiate + carbidopa: 1 (4) Nortryptiline: 1 (4)	0.58
**Indication for colonoscopy**					
Abnormal imaging	0 (0.0%)	1 (12.5%)	0 (0.0%)	1 (4.0%)	0.71
Diarrhea	1 (11.1%)	2 (25.0%)	2 (25.0%)	5 (20.0%)	
Hematochezia	6 (66.7%)	3 (37.5%)	3 (37.5%)	12 (48.0%)	
IBD	0 (0.0%)	0 (0.0%)	1 (12.5%)	1 (4.0%)	
Melena	1 (11.1%)	0 (0.0)%	1 (12.5%)	2 (8.0%)	
Abdominal pain	0 (0.0%)	1 (12.5%)	0 (0.0%)	1 (4.0%)	
Anemia	1 (11.1%)	1 (12.5%)	1 (12.5%)	3 (12.0%)	

**Fig. 1:**
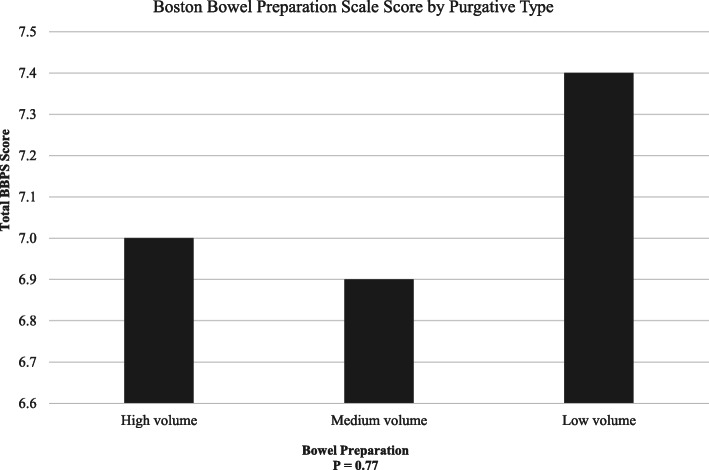
Relation between mean total Boston Bowel Preparation Scale Score (BBPS) and type of bowel preparation

Patients who received low volume preparation achieved a higher total BBPS score (mean 7.4, SD ± 1.62) than patients who received high volume preparation (mean total BBPS score 7.0, SD ± 1.41) and medium volume preparation (mean total BBPS score 6.9, SD ± 1.55), although the differences were not statistically significant (*P* = 0.77) (Fig. 1). Seven patients in the low volume group, eight in the high volume group, and six in the medium volume group had BBPS score ≥ 6 (*P* = 0.70), which is considered adequate colon preparation [12].

In the large volume group, mean BBPS score for right colon was 2.4 (0.52), for transverse colon was 2.5 (0.53) and for left colon was 2.3 (0.46). In the medium volume group, mean BBPS for right colon was 2.4 (0.74), for transverse colon was 2.5 (0.53) and for left colon was 2.0 (0.53). Finally, in the low volume group, BBPS score for right colon, transverse colon and left colon was 2.6 (0.53), 2.7 (0.76), and 2.1 (0.69), respectively. In all cases, cecal intubation was achieved. No procedures were delayed or cancelled due to poor bowel preparation.

**Fig. 2:**
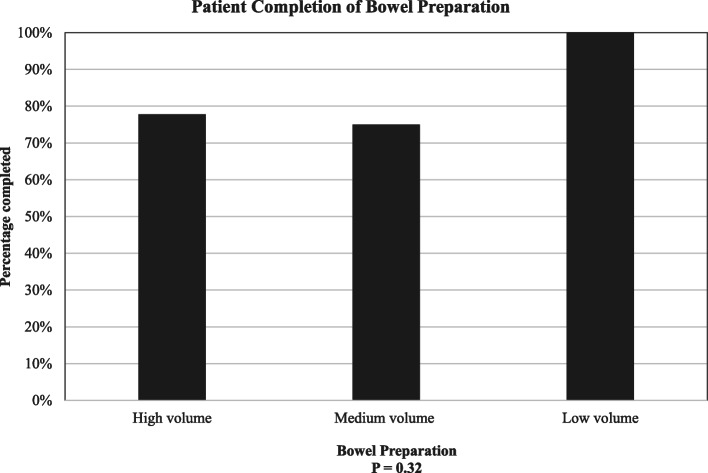
Ability to completely finish colon preparation among different colon preparation solutions

With regard to tolerance to colon cleansing, 100% of patients who received low volume preparation reported finishing bowel preparation completely, whereas 77.8% of the large volume group and 75% of the medium volume group reported accomplishment all purgative intake (*P* = 0.32), shown in the Fig. 2. Among patients who did not finish the preparation, two individuals receiving large volume preparation took 3920 and 1500 mL, and two patients receiving medium volume preparation took 1860 and 1800 mL.

The perception of unpleasant taste demonstrated a significant difference between the low volume group and high volume/ medium volume groups (*P* ≤ 0.01). Mean (SD) score for this adverse event reported by patients who had low volume preparation was 0.6 (0.74), while patients who had large volume preparation reported 2.2 (0.97) and medium volume group mean score was 2.1 (1.36). Other parameters analyzed were nausea and vomiting. Mean (SD) score for patient reported nausea was 0.9 (1.27), 0.5 (1.07), and 0 in the large volume, medium volume, and low volume groups respectively, and mean (SD) score for vomiting was 0.1 (0.33), 0 and 0 in the large volume, medium volume, and low volume groups respectively (Table 4).

**Table 4:** Patient reported adverse effects of bowel preparations

	**Large volume**	**Medium volume**	**Low volume**	***P*-value**
**Unpleasant taste**				
Mean (SD)	2.2 (±0.97)	2.1 (±1.36)	0.6 (±0.74)	≤ 0.01
Range	1–3	0–4	0–2	
**Nausea**				
Mean (SD)	0.9 (±1.27)	0.5 (±1.07)	0 (0.0)	0.19
Range	0–3	0–3	0	
**Vomiting**				
Mean (SD)	0.1 (±0.33)	0 (0.0)	0 (0.0)	0.43
Range	0–1	0	0	

## Discussion

Our pilot study is the first to compare the quality of bowel preparation based on volume in a prospective and randomized manner in hospitalized patients. Patients who received the low volume preparation showed a trend towards better BBPS score, compared to those receiving larger volume preparations, but that was not statistically significant. Importantly, low volume preparation was perceived to taste better to patients, which likely plays a factor in patient compliance. Additionally, the low volume preparation had lower rates of patient reported nausea and vomiting which also likely contributes to a higher rate of bowel preparation completion in this group.

A poor bowel preparation for colonoscopy has detrimental consequences, such as decreased identification of pathology, increased procedure time, and decreased rates of cecal and terminal ileum intubation. Inadequate bowel preparation also leads to shortened interval colonoscopy duration and increased health care costs, not to mention additional risks to the patient and increased time off of work than would be required otherwise [13, 14]. Furthermore, risk of adverse events such as colon perforation may be increased with inadequate colon cleansing [14]. These factors emphasize the importance of prospective studies reviewing the efficacy and quality of bowel cleansing, especially in hospitalized patient cohorts.

Predictors of inadequate bowel preparation for ambulatory colonoscopies cannot be extrapolated to the inpatient setting given the differences between inpatient and outpatient populations. These differences include distribution of age, level of physical activity, prevalence of comorbidities and indication for colonoscopy which are vastly different between the two populations [7].

Some authors have reported better results of inpatient colonoscopy preparation with split-dose administration of 4 L polyethylene glycol (PEG) [15], based on the outpatient data that shows that split-dose PEG solution is superior to single-dosing [8, 16, 17]. In the outpatient setting, it is known that timing between completion of purgative intake and the colonoscopy is an important factor for bowel-preparation quality, regardless if the procedure is performed in the morning or afternoon [9–11]. In our study, we used split dosing of bowel preparation in all subjects, giving half of the solution starting the evening prior to day of procedure and the remaining half in the morning of the planned procedure.

Physician, nursing, and patient education has also shown to be an efficient tool to optimize colonoscopy cleansing in the inpatient setting [18]. However, Chorev et al did not find significant improvement in preparation quality or in colonoscopy success in hospitalized patients after departmental institution of an educational program for healthcare providers [15, 18].

The standard preparation for patients with medical comorbidities of renal failure, congestive heart failure, or liver disease are 4 L PEG-electrolyte solutions [1, 2, 5]. Nonetheless, it is reported that 1 in 7 patients may not be compliant to a bowel preparation mainly due to the volume [19]. Improved results of preparation are achieved with better compliance, which has been shown to be related to decreased bowel preparation volume, palatability and regimen simplicity [2, 17, 19–21]. We found that 100% of subjects who received low volume preparation finished their bowel preparation completely, in opposition to medium volume preparation (75%) and high volume preparation (77.8%). Reasons for this difference may be explained by the lower volume and better perceived taste of the low volume preparation used in this study (*P* ≤ 0.01), both of which can enhance adherence of a colon cleansing regimen.

Our findings are consistent with a prospective study performed by Gu et al. with more than 4300 outpatient colonoscopies that reports better tolerability and cleansing with SuPrep® (low-volume regimen bowel preparation) than GoLYTELY® (high-volume preparation) [17].

A retrospective study by Corliss et al. described a 44.1% rate of inadequate bowel preparation (total BBPS < 7) among hospitalized patients receiving standard solution of GoLYTELEY® (large volume preparation), in comparison to a rate of 22.6% of inadequate bowel preparation among inpatients receiving SuPrep® (low-volume preparation) in split-dose fashion [22].

Our study has some limitations. While novel and prospective, one of the largest limitations to our study is our small sample size, which may affect the statistical significance and impairs any definitive conclusions. There is no FDA approved purgative for patients with some medical conditions as such as congestive heart failure, advanced renal disease, and decompensated liver disease; therefore the standard 4 L Polyethylene Glycol has been the default choice for such patients. Since the hospitalized population has more patients with these conditions, our findings may not be generalized to all inpatients and the potential for prescribing error in such patients should be considered. Additionally, these results are not generalizable to all other low volume preparations. Given the prospective nature of our study, all our patients were on a clear liquid diet the day prior to procedures, and this may not be the case for all hospitalized patient undergoing colonoscopies. This study cannot be generalized to very urgent colonoscopies that could require rapid preparation as split dose preparation would not be appropriate in this setting. Some patients in our study were currently using opioids, one patient was on nortriptyline and one patient was on Carbidopa; medications which may decrease intestinal motility. When evaluated these medication usages were not different among the preparation groups. Finally, the lack of information about prior inadequate bowel preparation along with subjective patient report about completion of preparation solutions in each group may have affected our results.

## Conclusions

In summary, we found that low volume colon preparation may be preferred in the inpatient setting due to its better rate of tolerability and comparable bowel cleanliness when compared to larger volume preparations, although we cannot overreach any definitive conclusion. Low volume preparation also demonstrated a lower rate of reported unpleasant taste and nausea than other medium and high volume preparations. This study highlights the fact that larger volume preparation may not be superior to low volume preparation in the inpatient setting and further more robust studies are required to confirm these findings.

## Data Availability

The datasets used and/or analyzed during the current study are available from the corresponding author on reasonable request.

## Abbreviation

BBPS: Boston Bowel Preparation Scale
